# Case Report: Multimodal Imaging of Toxic Retinopathies Related to Human Immunodeficiency Virus Antiretroviral Therapies: Maculopathy vs. Peripheral Retinopathy. Report of Two Cases and Review of the Literature

**DOI:** 10.3389/fneur.2021.663297

**Published:** 2021-06-17

**Authors:** Arthur Hammer, François-Xavier Borruat

**Affiliations:** Department of Ophthalmology, Hôpital Ophtalmique Jules-Gonin, Fondation Asile des Aveugles, University of Lausanne, Lausanne, Switzerland

**Keywords:** HIV, ritonavir, didanosine, retinopathy, case report

## Abstract

**Purpose:** We report two patients with toxic retinopathy from either ritonavir or didanosine and reviewed the literature on the topics. We provide an overview of the retinal toxicity of these two antiretroviral drugs in human immunodeficiency virus-positive patients.

**Methods:** First, we performed a retrospective study of the medical charts of two patients examined by us, one with ritonavir maculopathy and one with didanosine peripheral retinopathy. Secondly, we searched the world literature for similar cases through PubMed and Google Scholar, using the terms “HIV,” “AIDS,” “ritonavir,” “didanosine,” “maculopathy,” “retinopathy,” “visual loss,” and “toxicity” to retrieve the appropriate literature on the subject.

**Results:** Patient 1: A 49-year-old woman complained of progressive central visual loss over the past 12 months. History disclosed ongoing ritonavir therapy for the past 11 years. Ritonavir maculopathy was diagnosed, and visual loss increased relentlessly despite cessation of treatment. Patient 2: A 55-year-old man complained of slowly progressive peripheral visual field constriction for the past 5 years. History disclosed didanosine therapy for 13 years, however, stopped 4 years before the onset of visual symptoms. No alteration of therapy was offered to patient 2 as didanosine therapy was interrupted 9 years previously. Since 2011, 11 cases of ritonavir maculopathy have been reported in the literature. Relentless worsening of vision was reported in 3/7 patients despite cessation of ritonavir therapy. Didonasine peripheral retinopathy was first described in 1992, and a total of 24 patients have been reported since. Relentlessly progressive peripheral retinopathy was diagnosed despite the previous cessation of therapy in 14 patients.

**Conclusion:** Ritonavir causes a slowly progressive atrophic maculopathy, and didanosine toxicity results in a relentlessly progressing peripheral atrophic retinopathy. The relentless progression of both toxic retinopathies reflects permanent alterations of the retinal metabolism by these medications. Both ritonavir and didanosine toxic retinopathies are rare events, but their clinical presentation is highly specific.

## Introduction

Nowadays, patients affected by the human immunodeficiency virus (HIV) benefit from effective therapy, ensuring a majority of them a long-term remission. Current treatments of HIV are based on a combination of antiviral drugs, some of which can manifest undesirable secondary effects. Although rare, toxic retinopathy may result from the chronic use of antiviral drugs. Up to now, a few cases of toxic retinopathy have been reported, mostly associated with the chronic use of either ritonavir or didanosine. Ritonavir is associated with maculopathy, whereas didanosine toxicity manifests as mid-peripheral retinopathy (1–3). Visual loss is progressive, relentless, and usually permanent. Early recognition of such retinopathies is mandatory, as discontinuation of the medication may halt the progression of the retinopathy. We report two patients, one with ritonavir maculopathy and the other with didanosine peripheral retinopathy, and we summarize the world literature on the subject.

## Case Reports

### Case 1

A 49-year-old woman, HIV positive since 1993, was referred in 2018 for a progressive, painless visual loss starting 12 months ago. Her past neuro-ophthalmic history was remarkable for Candida endophthalmitis of her right eye, necessitating an enucleation in 2002. In 2008, she presented a left complete temporal hemianopsia resulting from right occipital toxoplasmosis; visual acuity (VA) was 20/25, and fundus examination was normal. HIV therapy consisted of a combination of daily lopinavir 400 mg, ritonavir 100 mg, abacavir 600 mg, and lamivudine 300 mg for the past 11 years. Compliance to therapy was poor, and, at the time of examination, she presented an elevated viremia (2,250 copy/ml) and a CD4 count at 190/mm^3^.

**Figure 1:**
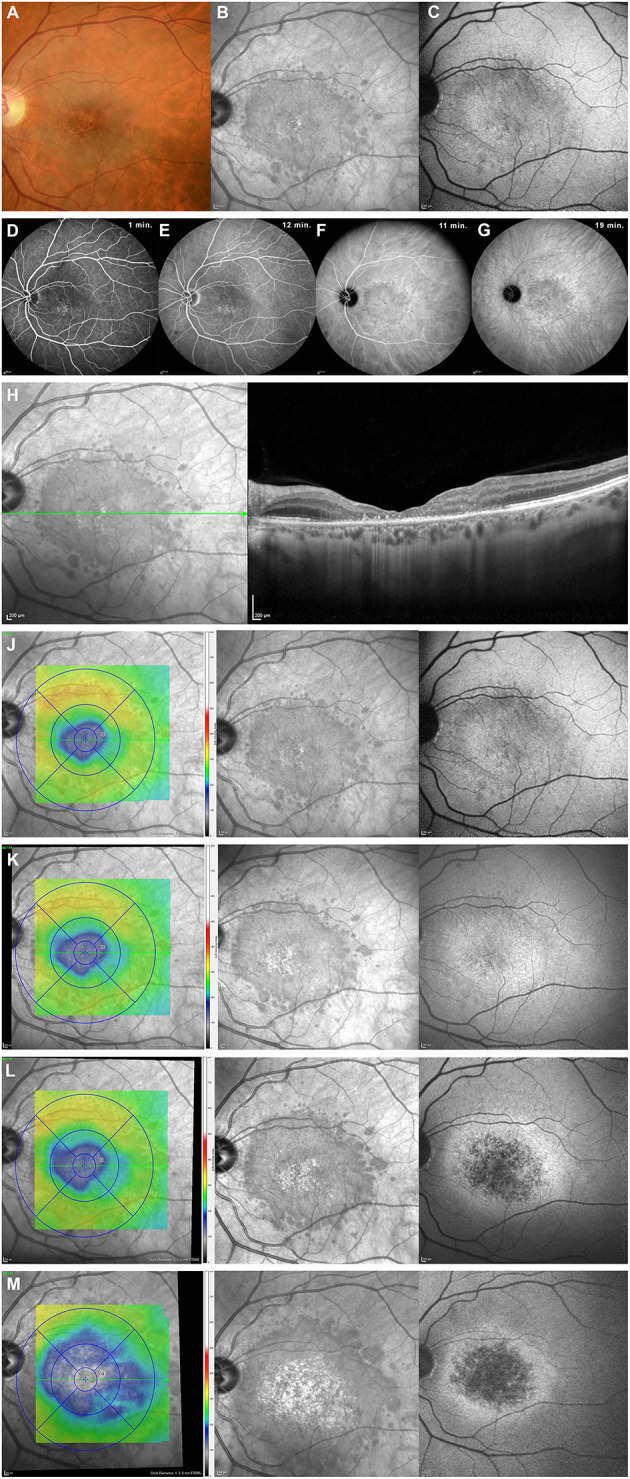
Multimodal imaging of ritonavir toxic maculopathy (patient 1). Top row—Initial examination of fundus showed discrete macular atrophy of granular appearance **(A)**. A wider extent of maculopathy was best demonstrated by pseudo-infrared photography **(B)**, which very precisely delineated borders of maculopathy. Autofluorescence fundus photography **(C)** was somehow less informative than pseudo-infrared technique. Second row—Retinal fluorescein angiography showed a mottled hyperfluorescent appearance of left macula, from early phase on **(D)**, without leakage in intermediate or late phases **(E)**, resulting from retinal pigment epithelium (RPE) window defects, without any other abnormalities. Indocyanine green angiography provided a more accurate anatomical evaluation of extent of maculopathy, which remained hypocyanescent centrally, bordered by a ring of small areas of stippled hypercyanescence during intermediate **(F)** and late phases **(G)**. Third row—OCT (Spectralis, Haag-Streit AG, Koniz, Switzerland) imaging of macula revealed a complete loss of external layers of retina with irregular stippled thickening of RPE **(H)**. Bottom rows **(J–M)**—Progression of maculopathy despite cessation of ritonavir. Each row depicts overall retinal situation at one point in time (**J** = initial, **K** = 1 month, **L** = 3 months, and **M** = 12 months after cessation of ritonavir). Left column represents global macular thickness (blue color represents thinning of retina), middle column shows results of pseudo-infrared photography, and right column exhibits results of autofluorescence. A progressive increase in both extent of maculopathy and an increase in retinal thinning is obvious.

In May 2018, the central visual function of her left eye was decreased with a VA of 20/400 and altered color vision (1/13 on Ishihara pseudo-isochromatic plates). Slit-lamp examination was unremarkable, and intraocular pressure was 14 mmHg. The visual field (VF) of her left eye showed a stable, dense temporal hemianopia. Fundus examination revealed discrete macular atrophy of granular appearance with areas of hypo- and hyperpigmentation (Figure 1A). Pseudo-infrared and autofluorescent imaging revealed a more extensive maculopathy than what appeared on fundus examination (Figures 1B,C). Fluorescein angiography disclosed a small area of mottled hyperfluoresence of the left macula due to retinal pigment epithelium (RPE) window defect, without any other abnormalities (Figures 1D,E). Indocyanine green angiography disclosed a larger macular area of hypocyanescence, remaining hypocyanescent in the late phase (Figures 1F,G). Macular optical coherence tomography (OCT) revealed complete loss of the external layers of the retina, irregular stippled thickening of the RPE, and intact inner retina from the inner nuclear layer to the inner limiting membrane (Figure 1H). No structural anomaly of the optic nerve was detected on OCT, and vascularization of both retina and choroid was preserved on OCT angiography (not shown).

The clinical presentation evoked a differential diagnosis between central areolar choroidal dystrophy and ritonavir toxic maculopathy. Central areolar choroidal dystrophy is an autosomal dominant hereditary maculopathy characterized by a slowly progressive visual loss and altered choroidal vascularization. In the absence of either positive family history or alteration of choroidal vascularization on OCTA, and in the presence of a rapid rate of progressive visual loss (from 20/25 to 20/400 over 12 months), we hence suspected the toxic effect of ritonavir on the macula. Ritonavir was stopped, and the new antiviral therapy consisted of abacavir, dolutegravir, and lamivudine. Despite the cessation of ritonavir, the extent of the maculopathy continued to worsen over the next 18 months (Figures 1J–M).

### Case 2

A 55-year-old man, HIV positive since 1983 and treated with tritherapy since 1993, was referred in 2018 for progressive, painless concentric restriction of his peripheral VF in both eyes. Past ophthalmic history was remarkable for recurrent episodes of anterior non-granulomatous uveitis successfully treated with topical steroids between 1983 and 1993, related to ankylosing spondylitis, which never required systemic treatment. Past medical history was also remarkable for hypothyroidism, chronic Reiter syndrome, and Hodgkin lymphoma, previously treated by thoracic radiotherapy. HIV was treated with a combination of antiretroviral medications for the past 25 years, namely with didanosine for 13 years (1996–2009). Current therapy consisted of dolutegravir, emtricitabine, and tenofovir. The patient's HIV viremia was under control (<2.0E1), and the CD4 count was 792/mm^3^.

**Figure 2:**
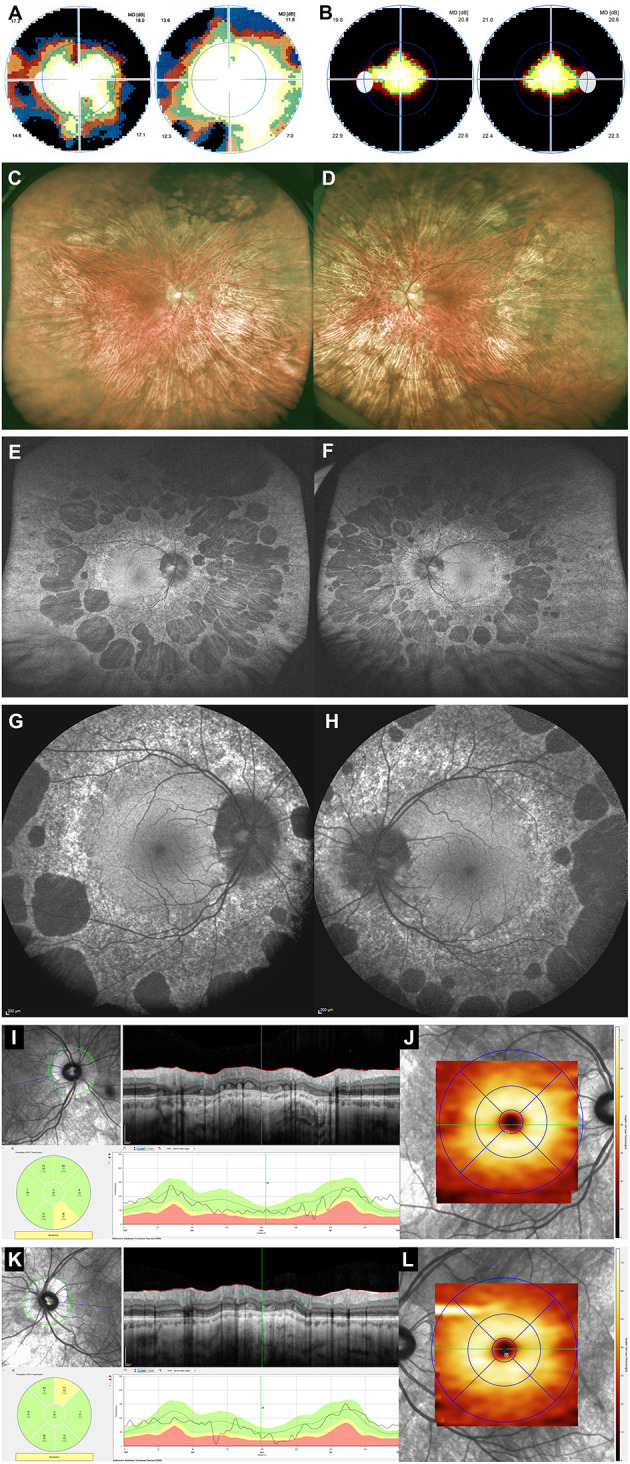
Multimodal imaging of didanosine peripheral retinopathy (patient 2). Top row—Results of computerized static perimetry (Octopus 300, program G1, Haag-Streit AG, Köniz, Switzerland) 5 years earlier **(A)** and at time of examination **(B)** revealed progressive peripheral constriction of visual field in both eyes. Second row—Widefield fundus photography (Optos, GmbH, Zweigneiderlassung Zug, Switzerland) showed a 360° of mid-peripheral areas of chorioretinal atrophy, sometimes coalescent, as well as pigment accumulation mainly in superior retina of both eyes **(C,D)**. Third row—Widefield autofluorescent photography (Optos, GmbH, Zweigneiderlassung Zug, Switzerland) better delineated shape and size of numerous mid-peripheral nummular areas of hypofluorescence in both eyes **(E,F)**. Fourth row—Posterior pole autofluorescence imaging (Spectralis, Haag-Streit AG, Koniz, Switzerland) revealed a normal macular area, peripapillary hypofluorescence, and a 360° perimacular ring of finely stippled hyperautofluorescence posterior to roundish areas of hypofluorescence **(G,H)**. Bottom row—OCT measurements (Spectralis, Haag-Streit AG, Koniz, Switzerland) of right eye **(I,J)** and left eye **(K,L)** revealed normal thicknesses for both RNFL **(I,K)** and retinal ganglion cell layer (RGCL) **(J,L)**.

The patient complained of slowly progressive peripheral visual loss for the past 5 years (Figure 2A). Visual acuity was decreased to 20/400 OU, and color vision was 0/13 OU on Ishihara pseudo-isochromatic plates. A functional non-organic component to the loss of VA was present, as the patient commented on small details of his own brain magnetic resonance imaging and kept on reading the same line when the distance examination was divided by four. The VF was severely constricted OU (Figure 2B). Anterior segment examination was unremarkable. Fundus examination revealed mid-peripheral roundish and well-delineated areas of chorioretinal atrophy, surrounded by hyperpigmentation, sparing the posterior pole (Figures 2C,D). Wide-field autofluorescent photography better delineated the peripheral punched-out round zones of chorioretinal atrophy (Figures 2E,F). Macular pseudo-infrared photography was unremarkable (not shown). Autofluorescence of the macula was normal but revealed a perimacular ring of fine hyperautofluorescent stippling (Figures 2G,H). Macular OCT and OCT angiography were unremarkable in both eyes (not shown), as were retinal nerve fiber layer and retinal ganglion cell layer thicknesses (Figures 2I–L). Peripheral OCT revealed complete loss of ellipsoid and thinning of RPE at the level of the atrophic lesions (not shown). A full-field electroretinogram revealed moderately severe rod dysfunction and moderate cone dysfunction (not shown). Fluorescein angiography showed peripheral window defect areas corresponding to the lesions readily visible during funduscopy but failed to show other abnormalities (not shown). A diagnosis of gyrate atrophy of the choroid was ruled out by normal blood levels of ornithine (65 μmol/L; normal values 30–100 μmol/L). Progressive outer retinal necrosis was unlikely, as the rate of visual loss was very slow, the CD4 count was normal, there was no macular involvement, and no retinal vasculitis was found on fluorescein angiography. Didanosine toxicity was diagnosed, although didanosine was stopped 9 years previously.

## Review of the Literature

We searched the world literature for similar cases through PubMed and Google Scholar, using the terms “HIV,” “AIDS,” “ritonavir,” “didanosine,” “maculopathy,” “retinopathy,” “visual loss,” and “toxicity.” Our literature search disclosed nine publications of retinal toxicity due to ritonavir between 2011 and 2020, for a total of 11 cases (1, 4–11). Details are provided in Table 1. Didanosine retinal toxicity was reported in 10 publications between 1992 and 2020, and 24 cases were reported (2, 3, 12–20). Details are provided in Table 2.

**Table 1:** Ritonavir maculopathy literature review.

**References**	**Age**	**Sex**	**Years of treatment**	**Daily dosage (mg)**	**Ritonavir treatment**	**VA RE**	**VA LE**	**Electrophysiology**	**Evolution after cessation**
Roe et al. (1)	46	M	1.6	200	Ongoing	20/40	20/160	NA	Worsened
	45	M	2.5	200	Ongoing	20/160	20/32	NA	Worsened
	40	M	5	200	Ongoing	20/120	20/400	NA	NA
Pinto et al. (4)	30	M	3	NA	Ongoing	20/70	20/70	Normal ERG	NA
Tu et al. (5)	47	M	7	200	Ongoing	20/25	20/400	NA	Improved
Non et al. (6)	36	M	14	NA	Ongoing	20/20	20/50	NA	Stable
Biancardi and Curi (7)	NA	NA	NA	NA	NA	NA	NA	NA	NA
Papavasileiou et al. (8)	59	M	8	100	Ongoing	20/50	20/32	Rod = Cone dysfunction	NA
Faure et al. (9)	49	M	10	200	Ongoing	20/125	20/100	Rod = Cone dysfunction	Stable
Mesquita et al. (10)	52	M	10	NA	Ongoing	20/630	20/630	NA	NA
Louie and Jones (11)	53	M	7	100	Ongoing	20/30	20/25	Rod = Cone dysfunction	Stable
Present Case, Case 1 (2021)	49	F	11	100	Ongoing	20/400	Enucleated	NA	Worsened

**Table 2:** Didanosine peripheral retinopathy literature review.

**References**	**Age**	**Sex**	**Years of therapy**	**Dosage (mg)**	**Didanosine therapy**	**VA RE**	**VA LE**	**Electrophysiology**	**Evolution after cessation**
Whitcup et al. (2)	7	F	0.88	NA	NA	20/20	20/20	NA	Stable
	2	F	0.75	NA	NA	20/20	20/20	NA	Stable
	8	M	1.6	NA	NA	20/20	20/20	NA	Stable
Whitcup et al. (3)	10	M	1.5	NA	NA	NA	NA	NA	NA
Nguyen et al. (12)	NA	M	2.65	275	NA	20/20	20/20	NA	NA
Whitcup et al. (13)	2	F	3.75	NA	NA	20/20	20/25	NA	Worsened
Cobo et al. (14)	32	M	2.1	400	Stopped 8 yrs ago	20/20	20/20	Abnormal ERG, abnormal EOG	Stable
	36	M	0.65	400	Stopped 1 month ago	20/20	20/20	Normal ERG, abnormal EOG	Stable
Muralha et al. (15)	32	F	NA	NA	NA	20/20	20/20		NA
Fernando et al. (16)	53	M	5	400	Ongoing	20/20	20/20	Rod > cone dysfunction	ERG improved
Pinto et al. (17)	50	F	NA	400	Stopped	20/20	20/25	Rod = Cone dysfunction	NA
Gabrielan et al. (18)	35	M	6	400	Stopped 2 years ago	20/25	20/25	Rod > cone dysfunction	Worsened
	52	M	NA	NA	Stopped	20/20	20/20	Rod > cone dysfunction	Stable
	54	M	4	NA	Stopped	20/70	20/80	Rod = Cone dysfunction	Stable
Haug et al. (19)	66	M	NA	NA	Ongoing	20/20	20/20	NA	NA
	42	M	11	NA	Stopped 12 years ago	20/20	20/20	NA	Worsened
	53	M	NA	NA	Stopped	20/25	20/25	NA	Worsened
	54	M	6	400	Stopped 14 years ago	20/30	20/30	NA	Worsened
	59	M	6.5	NA	NA	NA	NA	NA	NA
	53	M	5	NA	Stopped 5 years ago	20/30	20/25	NA	Stable
	45	M	6	NA	Stopped 4 years ago	20/30	20/25	NA	Worsened
	55	F	NA	NA	NA	20/25	20/25	NA	NA
	71	M	5.5	NA	Stopped 5 years ago	20/25	20/25	NA	Stable
Shifera and Singh (20)	52	F	8.5	400	Stopped 6.5 years ago	NA	NA	Rod & cone dysfunction	Stable
Present Case, Case 2 (2021)	55	M	13	NA	Stopped 9 years ago	20/400	20/400	Rod > cone dysfunction	Worsened

## Discussion

Nowadays, most HIV patients benefit from long-term remission due to the development of antiretroviral therapies. Frequently, it is a combination of various agents necessary to stabilize the course of the disease, and chronic therapy is needed. However, with longstanding therapies, undesirable effects may occur, and it may be a challenge to determine which medication is to be incriminated.

Our first patient presented a slow but relentless decrease of central visual function (visual acuity and color vision). Fundus examination revealed a mild granularity of the central macula, but autofluorescent imaging and, mostly, pseudo-infrared photographies disclosed very clearly a much more extensive degree of macular dysfunction. The differential diagnosis included either hereditary or toxic etiologies. We found no strong clinical argument to support a diagnosis of central areolar choroidal dystrophy. On the other hand, our patient shared several characteristics with previously published cases of ritonavir-induced maculopathy.

A review of the world literature disclosed 11 cases of ritonavir maculopathy reported between 2011 and 2019, to which we add our present case (patient 1). Results are compiled in Table 1 (1, 4–11). All reported patients were male, the only female being our patient. The age ranged from 30 to 59 years (46 ± 8.16 years), and maculopathy was found after 1.6 to 14 years of ritonavir therapy. Visual acuity ranged from 20/20 to 20/630 (median 20/70). Macular RPE changes were found in all but one patient and are reported as retinal pigment epitheliopathy, intraretinal crystalline deposits, foveal cysts, and macular telangiectasias. Data regarding the evolution of the maculopathy were available for 7/12 reported cases. The extent of the maculopathy in our patient was relentlessly increasing during the 18 months of follow-up (Figures 1J–M). Literature review revealed that three patients were reported as stable after cessation of therapy, whereas three others worsened after ritonavir therapy was halted. Only one patient improved relatively rapidly after cessation of ritonavir, and, interestingly, it was the only reported patient without detectable macular lesions by funduscopy or fluorescein angiography. Improvement after cessation of ritonavir may be possible if detection occurs very early on in the course of ritonavir maculopathy, as it occurred in this patient (5).

Ritonavir is an HIV protease inhibitor used in combination with other protease inhibitors because of its specific capacity to inhibit liver CYP3A4, which decreases the metabolism of other protease inhibitors. HIV protease inhibitors such as ritonavir and indinavir can increase retinal dehydrogenase activity and therefore increase the production of reactive oxygen species (21, 22). This could then lead to higher oxidative stress on the neuroretina. The macula, having high biochemical activity, could be more susceptible to an increase in reactive oxygen species, hence explaining the macular tropism of ritonavir toxicity. Furthermore, Vadlapatla et al. showed that ritonavir also displayed an anti-vasogenic effect by inhibiting both the expression and the secretion of vascular endothelial growth factor by retinal cells when exposed to hypoxia (23). This anti-vascular endothelial growth factor effect could also contribute to macular lesions during high metabolic demands by decreasing the feeding potential of a healthy retina.

Our second patient complained of slowly progressive constriction of his peripheral VF during at least 5 years (Figures 2A,B). Peripheral annular zones of RPE atrophy were found on funduscopy, and full-field ERG revealed a moderate to severe rod > cone dysfunction in both eyes. His visual acuity was severely decreased in both eyes, but a non-organic component was diagnosed as his OCT results were normal, and both his visual behavior and response to tests were not compatible with an organic cause. Initially, a differential diagnosis between hereditary, inflammatory/infectious, and toxic etiologies was entertained. Gyrate atrophy of the choroid was ruled out by normal serum levels of ornithine. The clinical presentation was not suggestive of progressive outer retinal necrosis. We hence retained didanosine as the causative agent of peripheral retinopathy despite cessation of didanosine therapy 9 years previously.

A world literature review (Table 2) disclosed 25 cases of didanosine retinopathy reported between 1992 and 2018, to which we add patient 2 of the present report (2, 3, 12–20). Their age ranged from 3 to 71 years, and there were 8 women for 17 men. Visual acuity ranged from 20/20 to 20/400 (median 20/20), being 20/25 or better in 34/42 eyes. Diagnosis of didanosine retinopathy was made during therapy for 7/20 patients and after didanosine that was stopped in 13/20 patients (1 month to 14 years after cessation of didanosine, median 5 years). Full-field ERG was performed in 6/25 cases and revealed moderate to severe dysfunction of both rods and cones. Our patient (patient 2) exhibited slowly progressive concentric VF restriction over 5 years, despite the withdrawal of didanosine 4 years before the onset of visual symptoms. Data on evolution after cessation of didanosine were available in 16/25 patients and showed stability of the retinopathy in 8/16 cases, worsening in 7/16 patients, and only one case of electrophysiological improvement (16).

Didanosine retinopathy is characterized by mid-peripheral well-delineated zones of RPE atrophy associated with relative loss of neurosensory retina and choriocapillaris. The macular area is generally intact. Overall, patients with didanosine retinopathy have a well-preserved visual acuity (median 20/20). Our patient (patient 2) presented a markedly diminished VA (20/400) despite a perfectly normal macula both on fundus and OCT examination (Figure 2). In his case, VA loss was attributed to a non-organic mechanism. Complete sparing of the macula was confirmed histopathologically by Whitcup et al. reported normal anatomy of the macular neurosensory retina, RPE cells, and choriocapillaris in a 6-year-old girl who had been treated with didanosine (13). They demonstrated atrophy of peripheral choriocapillaris, inner retina, and RPE layers. Using transmission electron microscopy, they showed the presence of membranous lamellar inclusions and cytoplasmic bodies in affected peripheral RPE cells (13). Retinal toxicity of didanosine is thought to result from primary insult to RPE cells and choriocapillaris.

Didanosine acts as a reverse transcriptase inhibitor through its property of adenosine nucleoside analog. Nucleoside analog reverse transcriptase inhibitors inhibit mitochondrial DNA (mtDNA) polymerase-γ, inducing subsequent mtDNA alteration (24). As suggested by Haug et al., didanosine chorioretinal lesions show phenotypical similarities (concentric chorioretinal atrophy) with retinopathies encountered in some mitochondriopathies (19). Wang et al. showed that didanosine leads to depletion of mtDNA and an increase in the quantity of mutated mtDNA (24). Recently, *in vitro* toxicity of didanosine on differentiated RPE cells has been studied by Hu X et al., revealing up to 60% depletion of mtDNA after 6–24 days of treatment (25). Whereas, they reported a potentially neuroprotective effect due to decreased oxidative stress potential, they hypothesized that with longer exposure to didanosine, the amount of mtDNA depletion would increase, causing permanent damage to RPE cells (25). Currently, no explanation has been found to explain why didanosine mtDNA toxicity particularly affects the peripheral retina, sparing the macular area. Permanent alteration of mtDNA could explain why didanosine peripheral retinopathy can progress despite cessation of therapy.

Ritonavir and didanosine, two drugs used in highly active antiretroviral therapy HIV treatment regimens, can lead to secondary undesirable chorioretinal toxicity with distinct patterns. Ritonavir toxicity results in a maculopathy, whereas didanosine toxicity will manifest as mid-peripheral concentric retinopathy, sparing the macular area. These specific patterns of retinal degeneration are not fully explained. Both ritonavir- and didanosine-toxic retinopathies can progress for several years despite cessation of therapy. This relentless progressive worsening of either type of retinopathy could be explained by either the irreversible binding of ritonavir to CYP3A4 or the persistent mtDNA damage induced by didanosine (24, 26).

The limitations of our study result mainly from its retrospective case report nature. There are neither blood nor paraclinical tests which could prove that those mentioned macular or peripheral retinopathies earlier resulted from the toxicity of either ritonavir or didanosine. However, the respective clinical presentation of our two patients corresponded to the previously reported cases of either ritonavir-maculopathy or didanosine-retinopathy, and other causes of retinopathy were either ruled out or very unlikely. We believe that the retinopathies of our two patients resulted from the toxic effects of either ritonavir or didanosine.

## Conclusion

HIV patients benefiting from long-term antiviral therapy are at risk of presenting various toxic retinopathies. Ritonavir toxicity will manifest as central visual dysfunction and a toxic maculopathy, which is best revealed with pseudo-infrared imaging and OCT. Didanosine toxicity will result in a progressive concentric VF loss due to a progressive well-delineated concentric mid-peripheral atrophic chorioretinopathy readily visible during funduscopy or with autofluorescent imaging.

Early diagnosis of such toxic retinopathies by the ophthalmologist is mandatory to try to prevent further visual loss.

## Summary

Although rare, toxic retinopathy may result from the chronic use of antiviral drugs. A few cases of toxic retinopathy have been reported, mostly associated with the chronic use of either ritonavir or didanosine. We report two patients, one with ritonavir maculopathy and the other with didanosine peripheral retinopathy. We also compiled the results of a review of the world literature on the subject.

## Data Availability Statement

The original contributions presented in the study are included in the article/supplementary material, further inquiries can be directed to the corresponding author.

## Ethics Statement

The studies involving human participants were reviewed and approved by CER Canton de Vaud, Avenue de Chailly 23, 1012 Lausanne, Switzerland. The patients/participants provided their written informed consent to participate in this study. Written informed consent was obtained from the individual(s) for the publication of any potentially identifiable images or data included in this article.

## Author Contributions

F-XB examined the patients, established the diagnosis, composed the Figures, and revised the manuscript. AH examined the charts and wrote the manuscript. Both authors contributed to the article and approved the submitted version.

## Conflict of Interest

The authors declare that the research was conducted in the absence of any commercial or financial relationships that could be construed as a potential conflict of interest.
